# Intact Type I Interferon Receptor Signaling Prevents Hepatocellular Necrosis but Not Encephalitis in a Dose-Dependent Manner in Rift Valley Fever Virus Infected Mice

**DOI:** 10.3390/ijms232012492

**Published:** 2022-10-18

**Authors:** Lukas Mathias Michaely, Lukas Schuwerk, Lisa Allnoch, Kathleen Schön, Inken Waltl, Pia-Katharina Larsen, Andreas Pavlou, Chittappen Kandiyil Prajeeth, Guus F. Rimmelzwaan, Stefanie C. Becker, Ulrich Kalinke, Wolfgang Baumgärtner, Ingo Gerhauser

**Affiliations:** 1 Department of Pathology, University of Veterinary Medicine Hannover, Foundation, 30559 Hannover, Germany; 2 Center for Systems Neuroscience, 30559 Hannover, Germany; 3 Institute for Parasitology, University of Veterinary Medicine Hannover, Foundation, 30559 Hannover, Germany; 4 Research Center for Emerging Infections and Zoonoses (RIZ), University of Veterinary Medicine Hannover, Foundation, 30559 Hannover, Germany; 5 Institute for Experimental Infection Research, TWINCORE, Centre for Experimental and Clinical Infection Research, a Joint Venture between the Helmholtz Centre for Infection Research and the Hannover Medical School, 30625 Hannover, Germany

**Keywords:** encephalitis, hepatic necrosis, mouse model, Rift Valley fever virus, type I interferon

## Abstract

Rift Valley fever (RVF) is a zoonotic and emerging disease, caused by the RVF virus (RVFV). In ruminants, it leads to “abortion storms” and enhanced mortality rates in young animals, whereas in humans it can cause symptoms like severe hemorrhagic fever or encephalitis. The role of the innate and adaptive immune response in disease initiation and progression is still poorly defined. The present study used the attenuated RVFV strain clone 13 to investigate viral spread, tissue tropism, and histopathological lesions after intranasal infection in C57BL/6 wild type (WT) and type I interferon (IFN-I) receptor I knockout (IFNAR^−/−^) mice. In WT mice, 10^4^ PFU RVFV (high dose) resulted in a fatal encephalitis, but no hepatitis 7–11 days post infection (dpi), whereas 10^3^ PFU RVFV (low dose) did not cause clinical disease or significant histopathological lesions in liver and the central nervous system (CNS). In contrast, IFNAR^−/−^ mice infected with 10^3^ PFU RVFV developed hepatocellular necrosis resulting in death at 2–5 dpi and lacked encephalitis. These results show that IFNAR signaling prevents systemic spread of the attenuated RVFV strain clone 13, but not the dissemination to the CNS and subsequent fatal disease. Consequently, neurotropic viruses may be able to evade antiviral IFN-I signaling pathways by using the transneuronal instead of the hematogenous route.

## 1. Introduction

Rift Valley fever (RVF) is a zoonotic disease caused by infection with RVF virus (RVFV), a Phlebovirus of the *Phenuiviridae* family [1,2,3]. This virus affects a wide variety of host species including ruminants and humans [2]. RVFV is endemic in large areas of the African continent as well as on the Arabian Peninsula; it causes major economic losses in ruminant livestock due to so called “abortion storms” in adults and up to 100% mortality rates in young animals [3,4,5]. Furthermore, RVF is classified as an emerging disease, representing a possible threat for Europe and other continents [6,7]. Approximately 1% of RVFV infected human patients develop severe clinical symptoms including hemorrhagic fever, liver failure or long-term neurologic sequelae occurring weeks to months after the initial disease [1,2,8]. Given these severe consequences in humans and the constantly exceeding area of endemic occurrence, scientific investigation of RVF is mandatory [1,9].

One manifestation affecting RVF patients is hepatic disease, resulting in jaundice and hemorrhagic fever [3,5]. This rather rare manifestation (1–2% of human infections) of RVF leads to high fatality rates and patients usually succumb to the disease within two weeks after the onset of symptoms [2,10]. Hepatitis occurs due to the epithelial, especially hepatocellular tropism of RVFV [11,12,13,14]. After initial replication in cutaneous cells, including dendritic cells, which are entered via dendritic cell-specific intercellular adhesion molecule-3-grabbing non-integrin (DC-SIGN)-mediated endocytosis, the virus spreads via the blood stream and infects hepatocytes among other epithelial tissues [12]. Due to the limited expression of DC-SIGN in hepatocytes, the infection occurs via the DC-SIGN homologue liver/lymph node-specific intercellular adhesion molecule-3-grabbing non-integrin (L-SIGN), which is present in liver sinusoidal epithelial cells, heparan sulfate mediation, or the scavenger receptor class A member I (SCARA-I), that also has been shown to play a role in the uptake of adenovirus 5 and herpes simplex virus [11,13,15,16,17].

Another rare, but critical, complication in humans is late onset encephalitis, which accounts for up to 17% of severely affected RVF patients [8]. This RVF encephalitis (RVFE) occurs up to 60 days after the initial disease and can lead to severe lifelong impairment or even fatal outcome in every second patient [1,5,8]. Hitherto, detailed pathomechanisms of this disease, including virus entry into the central nervous system (CNS), have not been elucidated entirely [1,9,13,18]. Previous studies showed that aerosol infection leads to more severe and earlier development of RVFE in mice than infection via intraperitoneal or subcutaneous injection [19]. Similarly, increased severity of RVF-associated disease after aerosol exposure has been reported in humans [19,20,21,22,23]. Large amounts of virus have been detected along the olfactory epithelium and the olfactory bulb in rats and mice that were intranasally infected [20,24]. Other mechanisms of neuroinvasion, including penetration of the blood–brain barrier (BBB) or travel via neuromuscular junctions, seem to be less likely [20]. Nevertheless, viral entry through the BBB has been described as a late pathogenic event during RFVE in rats [25]. In mice, RVFV causes neuronal necrosis, microhemorrhages and a meningoencephalitis with marked infiltration by lymphocytes and macrophages forming perivascular cuffs like human CNS lesions [24,26,27,28,29].

However, the detailed pathogenesis of both RVF hepatitis and encephalitis has not been elucidated so far, especially regarding the involvement of humoral and cellular immune response mechanisms [9,21,24,26,30,31]. Studies in humans are rare and mainly limited to case reports during larger outbreaks [27,29,32]. Due to the large variety of susceptible hosts, a broad spectrum of animal models including primates, ferrets, and rodents have been used to investigate RVF [26,33,34,35,36]. Of these models, mice are of special interest because they exhibit a biphasic disease that resembles the late occurrence of human RVFE [20,31]. Mice typically develop a fulminant hepatitis during their first phase of disease, which often leads to a lethal outcome prior to any neurologic complications [24,31,34,35]. However, in case of survival, a subsequent encephalitis beginning at 6 days post infection (dpi) occurs [24,31,34,35]. Modification of susceptible mouse strains allowed to demonstrate the important role of type I interferon (IFN-I) signaling for early disease control, while cells of both the innate and adaptive immune system are crucial for virus clearance [37]. Thus, gain of information on the clinical course, viral spread and tissue tropism of RVFV in both wild type (WT) and immunocompromised animals is crucial to shed light on the detailed pathogenesis of RVF.

The IFN-I signaling pathway plays an essential role in the innate immune response during viral infections [18,38]. Mice lacking the IFN-I receptor I (IFNAR) exhibit a high susceptibility towards intraperitoneal infection with even attenuated RVFV strains highlighting the importance of IFNAR signaling during RVFV infection [39,40,41,42]. Thus, the hypothesis of the present study is that the IFN-I response is a crucial determinant of the outcome of intranasal RVFV infection.

Therefore, the aim of the present study was to characterize the viral dissemination and tissue tropism of RVFV in C57BL/6 wild type (WT) and IFNAR-deficient C57BL/6 (IFNAR^−/−^) mice upon intranasal infection with RVFV.

## 2. Results

### 2.1. Survival and Clinical Signs

**Figure 1:**
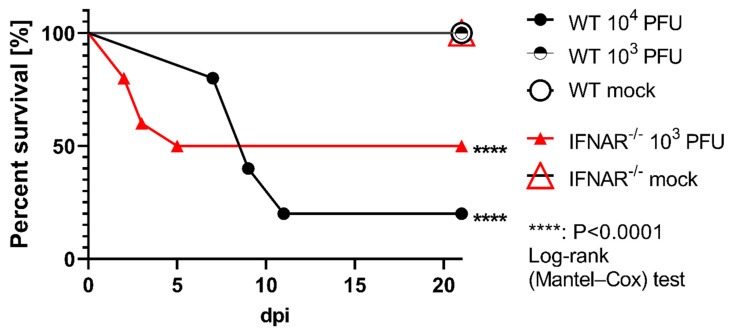
Survival of wild type (WT) and type I interferon receptor deficient (IFNAR^−/−^) mice infected with Rift Valley fever virus (RVFV). All mock-infected WT and IFNAR^−/−^ mice, as well as WT mice infected with 10^3^ plaque-forming units (PFU) RVFV, survived until 21 days post infection (dpi). Five out of ten IFNAR^−/−^ mice infected with 10^3^ PFU RVFV, and four out of five WT mice infected with 10^4^ PFU RVFV exhibited severe disease and died or had to be euthanized until 5 (IFNAR^−/−^) and 11 dpi (WT).

IFNAR signaling is of pivotal significance to restrict several viruses with neurotropic potential. In order to delineate the role of IFNAR signaling during RVFV infection in encephalitis prevention, mice were infected intranasally with RVFV. This infection route was chosen because it has been shown that many other viruses gain CNS entry via olfactory sensory neurons. Four out of five WT mice infected with 10^4^ PFU developed severe clinical signs comprising hind limb paresis, hunched posture, and paralysis and died or had to be euthanized 7–11 dpi (Figure 1). Therefore, the infection dosage was downscaled to 10^3^ PFU resulting in no clinical signs in WT mice, whereas five out of ten IFNAR^−/−^ mice infected with 10^3^ PFU demonstrated a rapid course of disease and died within 2–5 dpi. These data suggest that IFNAR signaling significantly protects the host from RVFV infection.

### 2.2. Histology

**Figure 2:**
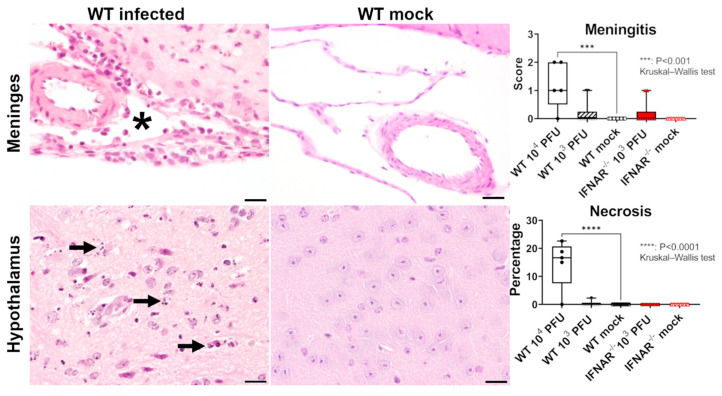
Histological findings in the brain of mice infected with Rift Valley fever virus (RVFV). Moderate lymphohistiocytic leptomeningitis (asterisk) in a wild type (WT) mouse at 11 days post infection (dpi) with 10^4^ plaque-forming units (PFU) RVFV. Hypothalamic neuronal necrosis (arrows) in a WT mouse at 11 dpi with 10^4^ PFU RVFV. No lesions in the meninges and hypothalamus of a mock-infected control animal sampled at 21 dpi. Hematoxylin and eosin staining, Bars = 20 µm. Graphs show the semiquantitative scores of meningitis and the percentage of area with necrosis in the investigated groups. Box plots with all data points. IFNAR^−/−^: type I interferon receptor knockout mice.

**Figure 3:**
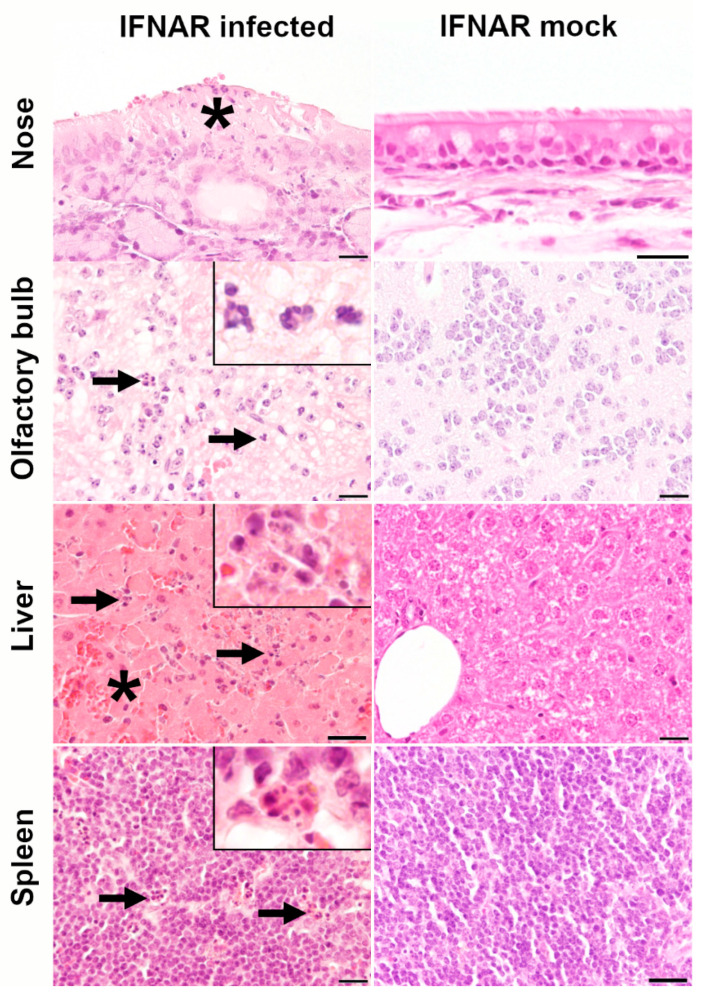
Histological findings in the olfactory epithelium, olfactory bulb, liver, and spleen of type I interferon receptor knockout (IFNAR^−/−^) mice infected with Rift Valley fever virus (RVFV). Infected mice are shown in the left column and mock-infected control mice in the right column. Mild necrosuppurative inflammation of the respiratory mucosa (asterisk) in an IFNAR^−/−^ mouse at 5 days post infection (dpi) with 10^3^ plaque-forming units (PFU) RVFV. Mild neuronal necrosis (arrows; inset) in the olfactory bulb of an IFNAR^−/−^ mouse at 2 dpi with 10^3^ PFU RVFV. Moderate hepatocellular necrosis (arrows; inset) and hemorrhage (asterisk) in the liver of an IFNAR^−/−^ mouse at 5 dpi with 10^3^ PFU RVFV. Multifocal lymphocytolysis (arrows, inset) in the spleen of an IFNAR^−/−^ mouse at 3 dpi with 10^3^ PFU RVFV. No lesions were present in mock-infected control animals sampled at 21 dpi. Hematoxylin and eosin staining, Bars = 20 µm.

To dissect the immunopathological events associated with RVFV infection, CNS lesions were detected in the brain (n = 4, 80%) and spinal cord (n = 2, 20%) of four WT animals infected with 10^4^ PFU that died at 7–11 dpi (80%), consisting of multifocal neuronal necrosis (n = 4) of varying severity and mild (n = 2) to moderate (n = 2) leptomeningitis (Figure 2). In these animals, neuronal necrosis was observed in all examined brain regions except the cerebellum, with consistently moderate-to-severe alterations in the hypothalamus with up to 55% of the affected area. In the one surviving WT mouse (10^4^ PFU), no alteration was observed in the brain at 21 dpi. In WT mice infected with 10^3^ PFU (n = 10), lesions were restricted to a focal mild leptomeningitis consisting of only sporadic infiltrating lymphocytes in the meninges of two animals. No deposition of extracellular matrix was detected using azan and picrosirius red special stainings in brain, spinal cord, or liver. In addition, no formation of perivascular edema was observed within alcian blue stained brain and spinal cord tissue, confirming the integrity of the vascular compartment. In contrast to WT animals, IFNAR^−/−^ mice infected with 10^3^ PFU RVFV that died until 5 dpi (n = 5, 50%) exhibited hepatic lesions, characterized by moderate to severe multifocal to coalescing hepatocellular necrosis and mild to moderate hemorrhages (Figure 3). A small area of neuronal necrosis was observed in the olfactory nerve cell layer in the olfactory bulb of one IFNAR^−/−^ mouse that died 2 dpi. Two animals had a focal mild lymphohistiocytic leptomeningitis. Another IFNAR^−/−^ mouse exhibited a mild multifocal necrosuppurative rhinitis. Moreover, all diseased IFNAR^−/−^ mice showed a mild lymphocytolysis in the spleen and lymph nodes (overview given in Table 1). In both WT and IFNAR^−/−^ mice, the remaining organs showed no relevant microscopic lesions.

### 2.3. Immunohistochemistry and Immunofluorescence

**Figure 4:**
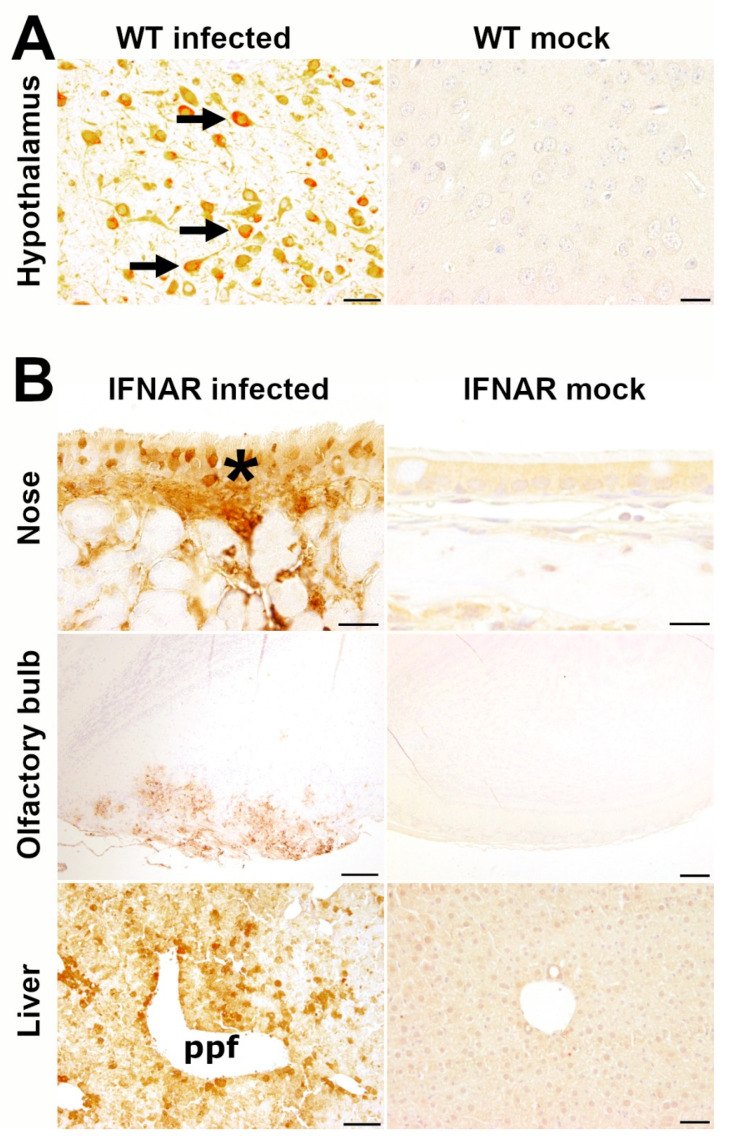
Rift Valley fever virus (RFVF) antigen in the hypothalamus of wild type (WT) mice (**A**) and nose, olfactory bulb and liver of type I interferon knockout (IFNAR^−/−^) mice (**B**). Infected mice are shown in the left column and mock-infected control mice in the right column. RVFV antigen was found in hypothalamic neurons (arrows) of a WT mouse infected with 10^4^ plaque-forming units (PFU) RVFV at 7 days post infection (dpi) as well as in the respiratory epithelium (asterisk) of the nose (5 dpi, same animal as in Figure 3), neurons of the olfactory bulb (2 dpi, same animal as in Figure 3) and hepatocytes (ppf: periportal field, 2 dpi) of IFNAR^−/−^ mice infected with 10^3^ PFU RVFV. Virus antigen was absent in all investigated organs of mock-infected control animals sampled at 21 dpi. Immunohistochemistry against RVFV nucleoprotein using the avidin-biotin-complex (ABC) method with 3,3′-diaminobenzidine tetrahydrochloride (DAB) as chromogen. Bars: hypothalamus and nose = 20 µm; olfactory bulb = 100 µm; liver = 50 µm).

To delineate the viral tropism of RVFV infection, both mock- and RVFV-infected mice (n = 45) were screened for RVFV antigen by immunohistochemistry. Immunopositive signals characterized by strong cytoplasmic and axonal immunolabeling were found in neurons of the brain (n = 4; 80%) and spinal cord (n = 2, 40%) of WT mice infected with 10^4^ PFU (Figure 4A and Figure S1). In the four WT animals observed with clinical disease, viral antigen was present in a high number of neurons throughout the brain, including cerebellar nuclei. In the two mice that showed lesions of the spinal cord seen in HE-staining, RVFV nucleoprotein was detected in the spinal cord accordingly. In the surviving WT mouse (10^4^ PFU), RVFV was not detected.

In IFNAR^−/−^ mice, a large amount of RVFV antigen was present in hepatic lesions of the five animals (50%) that died until 5 dpi (Figure 4B). Immunohistochemistry also revealed a high number of immunopositive leukocytes in the spleen (Figure S1), lymph nodes, intravascular monocytes (including those of the brain), and hematopoietic precursor cells in the bone marrow in these animals. In the one IFNAR^−/−^ mouse with necrosuppurative rhinitis, RVFV antigen was detected in several epithelial cells of the respiratory mucosa and single epithelial cells in the olfactory mucosa of the nose. Moreover, few immunopositive neurons were found in the olfactory nerve cell and adjacent glomerular layer of the olfactory bulb in the one IFNAR^−/−^ mouse that showed cellular necrosis in this area (Figure 4B). RVFV antigen was not detected in the brain or other tissues of all five surviving IFNAR^−/−^ mice or any mock-infected WT or IFNAR^−/−^ mouse (Table 1).

CD3 staining confirmed the presence of T cells in the leptomeninges of WT mice infected with 10^4^ PFU (Figure S2), whereas CD45R^+^ B cells were not detected using immunohistochemistry. GFAP and Iba-1 immunohistochemistry revealed no astrogliosis or microgliosis in the brain of WT and IFNAR^−/−^ mice after RVFV infection. Cleaved caspase-3 was found as an apoptotic marker in association with areas of necrosis in the brains of the four diseased WT animals, as well as in the olfactory bulb of the IFNAR^−/−^ mouse that showed corresponding lesions in HE-staining. Moreover, cleaved caspase-3 was found in the CNS of diseased WT mice and in the liver of the five diseased IFNAR^−/−^ mice in association with necrotic foci (Figure S3). Laminin and entactin/nidogen-1 immunohistochemistry did not show a deposition of basement-membrane associated extracellular matrix molecules in the lesioned brains and spinal cords suggesting an intact blood–brain barrier.

**Figure 5:**
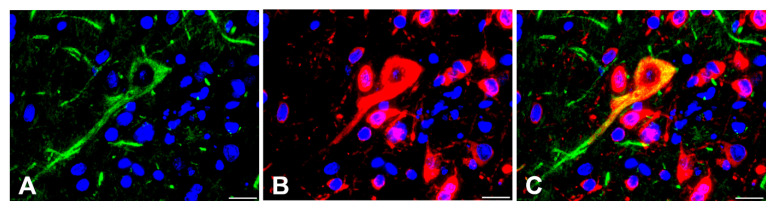
Double-labeling immunofluorescence for non-phosphorylated neurofilament (nNF) and Rift Valley fever virus (RVFV) nucleoprotein in the quadrigeminal lamina of a wild type of mouse at 9 days post infection with 10^4^ plaque-forming units RVFV. (**A**): Illustration of nNF in the neuronal soma and processes (Cy2-conjugated, green). (**B**): Visualization of RVFV antigen (Cy3-conjugated, red). (**C**): Superimposed signals of nNF and RVFV demonstrate the presence of RVFV antigen in the soma and axon of a neuron. Nuclei are stained with bisbenzimidine (blue). Bars = 20 µm.

Given that, in addition to the neuronal perikaryon, many neuronal processes in the brain were multifocally positive for RVFV in IHC staining, double-staining immunofluorescence for RVFV and non-phosphorylated neurofilament (nNF) was performed to verify transaxonal, viral spread. In WT mice (10^4^ PFU), double-labeling immunofluorescence for nNF and RVFV nucleoprotein confirmed the presence of RVFV antigen in neuronal processes (Figure 5) of all brain regions.

### 2.4. Reverse Transcription-Quantitative Polymerase Chain Reaction (RT-qPCR)

**Figure 6:**
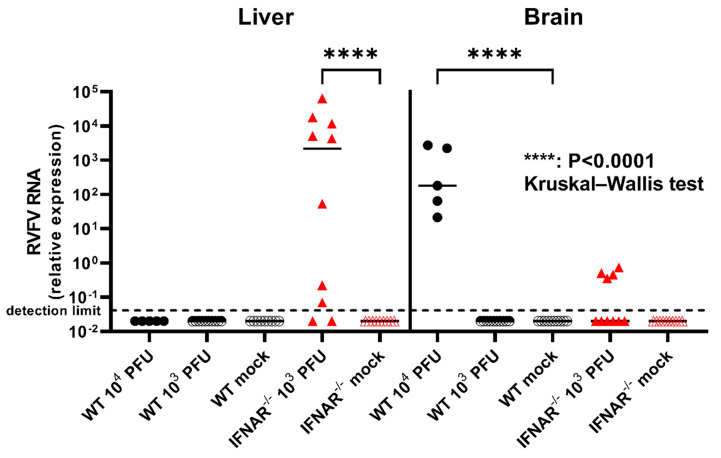
Reverse transcription-quantitative polymerase chain reaction (RT-qPCR) of liver and brain of wild type (WT) and type I interferon receptor knockout (IFNAR^−/−^) mice. In all five WT mice infected with 10^4^ plaque-forming units (PFU) Rift Valley fever virus (RVFV) viral RNA was detected in the brain at 7–21 days post infection (dpi), whereas viral RNA was present in the liver of eight out of ten IFNAR^−/−^ mice infected with 10^3^ PFU RVFV (2–21 dpi). Note low amount of viral RNA in the brain of four IFNAR^−/−^ mice with fatal disease (2–5 dpi). Relative expression of RVFV RNA was calculated by synchronal detection of β-actin as housekeeping gene. Shown are all data points with median values (lines). Kruskal–Wallis test with Dunn’s correction: **** *p* < 0.0001.

To investigate the viral load and pathogen dissemination in RVFV-infected mice, RT-qPCR of tissue samples from different organs was performed. The WT mice that were infected with 10^4^ PFU, four of which (4/5, 80%) had to be euthanized due to severe disease at 7–11 dpi, exhibited high viral loads in the brain, whereas virus was absent in the liver (Figure 6). No viral RNA was detected in the brains and livers of WT mice infected with 10^3^ PFU. On the contrary, all five IFNAR^−/−^ mice that were infected with 10^3^ PFU and succumbed to infection 2–5 dpi, exhibited high loads of RVFV RNA in the liver and comparatively low amounts of viral RNA were detected in the brains of four of these animals (Figure 6). The five IFNAR^−/−^ mice that survived until 21 dpi exhibited no virus within the brain, but three of these animals exhibited comparatively low amounts of viral RNA in their livers. No viral RNA was detected in the brains and livers of mock-infected control animals from both mouse strains.

## 3. Discussion

The objective of the present study was to evaluate the pathogenesis and viral spread of RVFV after intranasal instillation of C57BL/6 WT and IFNAR^−/−^ mice with RVFV clone 13.

Only treatment of WT mice with the high infection dose (10^4^ PFU) resulted in severe encephalitis in absence of alterations in other organ systems and led to death between 7 and 11 dpi. In contrast, low dose infection (10^3^ PFU) of IFNAR^−/−^ mice was sufficient for hematogenous viral spread and the development of severe hepatocellular necrosis without the appearance of fatal encephalitis resulting in death between 2–5 dpi. IFNAR^−/−^ mice cannot mediate signals via the IFN-I receptor, preventing IFNAR signaling, which regularly inhibits viral mRNA translation, induces RNA degradation, and activates cytotoxic T cells that eliminate infected cells [43]. This impairment highly increases their susceptibility even towards attenuated virus strains such as RVFV clone 13, thereby providing a possibility to investigate the pathogenesis of RVF [31,39]. The attenuation of RVFV clone 13 is based on the truncated nonstructural protein S (NSs), which is rapidly degraded by the proteasome [44,45]. RVFV NSs is an IFN-I antagonist that also inhibits cellular protein synthesis, induces chromosome segregation defects during mitosis, and triggers post-transcriptional downregulation of double-stranded RNA-activated protein kinase R (PKR) [39,40,46,47,48]. Correspondingly, an intraperitoneal infection of PKR^−/−^ mice with 10^5^ PFU (but not 10^3^ PFU) of RVFV clone 13 results in death at 5–6 dpi with high viral load in the liver, highlighting the antiviral effect of PKR [46].

Aerosol infection results in a more severe formation of RVFE in animal studies, which offers an opportunity to investigate its pathogenesis [19,49]. This is of particular interest with respect to human infections, the majority of which are most likely to happen via aerosol inhalation in close contact with infected animals [5,50]. The pathogenesis and the viral spread during RVFE are not completely understood; however, an ascending infection over the olfactory epithelium has been shown in the rat model [9,20]. This puts RVFV in line with various other viral agents like influenza A virus, herpesviruses, or paramyxoviruses that may use this route for a CNS infection [51]. Whether this ascension of RVFV is due to anterograde intraaxonal spreading or due to an intracanalicular spread via channels formed by olfactory ensheathing cells remains unclear [20]. An ascending infection via the olfactory route, as has been suggested in the rat model, is supported by the results due to the presence of RVFV-immunopositive cells in the olfactory mucosa of one IFNAR^−/−^ mouse and the detection of RVFV only in the olfactory bulb but not in other brains areas of another IFNAR^−/−^ mouse that died 2 dpi. Thus, the olfactory bulb likely represents the starting point of a fully developed RVFE seen in WT animals at later time points (7–11 dpi) [24,52]. Since no viral antigen was detected in the nose of most animals that were sampled at later time points, the olfactory epithelium apparently serves only as a transient layer of infection of this primarily hepato- and neurotropic virus, a finding that has been proposed previously [20].

The activation and proliferation of microglia in the olfactory bulb is necessary to limit viral spread within the CNS [53]. Interestingly, this gatekeeper function of resident microglial cells depends on an intact IFNAR signaling of astrocytes and neurons, which are the most important IFN-I producers during viral encephalitis [54,55]. Moreover, early activation of IFNAR signaling in the glomerular layer of the olfactory bulb seems to be critical for the control of viral replication [38]. The IFN-I response and cytokine expression of RVFV-infected microglia are largely dependent on RIG-I-like receptors (RLR) signaling via mitochondrial antiviral-signaling protein (MAVS), whereas RNA sensing by toll-like receptors (TLRs) only plays a minor role [56]. MAVS^−/−^ mice intranasally infected with 5x10^5^ PFU of the RVFV MP12 vaccine strain showed high levels of viral RNA inside the brain, which was associated with increased T and NK cell infiltration but an impaired T cell activation [56]. Functional NK cells, macrophages and lymphocytes seem to be essential for RVFV clearance [37]. In general, IFNAR^−/−^ mice have defects in T and NK cell development and functions impairing their adaptive antiviral immunity [57,58,59]. Due to the low infection dose and subsequent lack of virus in the CNS, lymphocytes were rarely detected in the brains of the present IFNAR^−/−^ mice. A higher dose of RVFV clone 13 is able to overwhelm antiviral immune responses in the olfactory bulb and induce RVFE, despite the truncation of its IFN-I response inhibiting NSs protein. While 10^4^ PFU of RVFV clone 13 caused lethal encephalitis in 80% of the infected C57BL/6 mice and a low amount of viral RNA was still present in the mouse that survived until 21 dpi, lesions were absent in WT mice infected with a ten times lower dose (10^3^ PFU) and virus was eliminated. Similarly, an intranasal infection with 10^4^ TCID_50_ but not 10^2^ TCID_50_ of a recombinant RVFV lacking NSs is still capable to induce lethal meningoencephalitis at 7–9 dpi in C57BL/6 mice, whereas the virus only causes a subclinical systemic infection when administered subcutaneously [21]. No IFNAR^−/−^ mouse that developed fatal liver disease survived longer than 5 dpi, preventing the development of RVFE. Moreover, surviving IFNAR^−/−^ mice lacked viral RNA at 21 dpi similar to the low dose infection of WT mice, which is likely attributed to the marked attenuation of RVFV clone 13 [39]. Interestingly, in a previous study only 50% of STAT-1^−/−^ mice developed disease after intranasal infection with 1.58 x 10^6^ TCID_50_ of the attenuated MP12 strain, showing that immunodeficient mice vary in their immune response to RVFV infection [31].

Once arrived in the CNS, viral spread within this compartment seems poorly restricted, since RVFV was found in all brain areas as well as the spinal cord of affected animals in this study. Double-labeling confirmed the presence of RVFV antigen in axons indicating intracerebral axonal spreading, a process described for several neurotropic viruses of the *Herpesviridae*, *Rhabdoviridae*, *Flaviviridae,* and *Picornaviridae* families and suggested for RVFV, too [20,60]. The independent development of RVFE without systemic lesions is enabled by the blood–brain barrier that breaks relatively late during RVFE [25]. Apparently, the intact enclosure of the brain limits infiltration of peripheral immune cells regardless of the systemic IFN-I response similar to other neurotropic viruses such as herpes simplex virus that display viral persistence within the CNS [61]. The late succumbence of WT animals (7–11 dpi), despite intracerebral RVFV detection already at 2 dpi, matches the long-term development of lethal encephalitis [19]. On the contrary, an unrestricted viral replication and hematogenous spread due to deficient antiviral immune mechanisms explains the rapid death due to hepatitis in IFNAR^−/−^ mice at 2–5 dpi. It cannot be ruled out that viral spread to other organs, e.g., kidneys or digestive system, contributes to this outcome of infection. However, the investigated sample tissues were chosen in accordance with previous literature reports of main RVF-affected organs [2,13,24].

Even after intranasal infection, IFNAR^−/−^ mice developed severe hepatocellular necrosis, whereas infection of WT mice with a higher dosage caused RVFE but no lesions in the liver or other organ systems. These findings support that IFNAR signaling plays a key role in determining RVF manifestation, regardless of the route of infection. Due to the truncated NSs protein, RVFV clone 13 induces high titers of IFN-I in infected animals that prevents systemic viral spread of this low virulent virus variant. As shown by the results, the infection of immunocompromised mice with a low virulence RVFV isolate can be used to study the pathogenesis of natural infection with virulent strains. This infection model can be used under biosafety level (BSL) 2 conditions and well mimics the natural infection of immunocompetent mice with wild type isolates of RVFV (BSL 3 organisms) that also leads to severe clinical signs due to fatal encephalitis and hepatitis.

The IFN-I response serves as the main immune response towards RVFV infection and thus, in IFN-I competent hosts RVFV clone 13 is regarded as attenuated after injection. However, in IFNAR^−/−^ mice that are unable to induce this protective mechanism, the NSs mutation does not prevent disease development as highlighted by the results. Although the intranasal infection route leads to an infection of olfactory epithelial cells, an initial leukocyte-associated viremia enables RVFV to target hepatocytes and cells of the reticuloendothelial system in IFNAR^−/−^ mice [2,24]. In the present study, five out of ten IFNAR^−/−^ mice, which are more than 20-fold backcrossed to the C57BL/6 background, survived an intranasal infection with 10^3^ PFU of RVFV clone 13, whereas other investigations showed that only 1–5 PFU of RVFV clone 13 killed 50% of IFNAR^−/−^ mice derived from an inbred 129SV/Ev genetic background [39]. As controversial reports exist on the survival probability of IFNAR^−/−^ animals in low and high virulent RVFV isolates, a mortality rate of less than 100% in IFNAR^−/−^ animals in the present study was not totally unexpected [39,62]. Moreover, the resistance to RVFV seems to be strongly influenced by the specific genetic background of the animals used for infection, an observation described in both RVFV-infected mice and rats [31,63].

In summary, the present study characterized RVFV clone 13 induced lesions after intranasal infection of IFNAR^−/−^ mice in detail and illustrated RVFV antigen in axons, thereby providing a BSL-2 usable mouse model of RVFE. Although RVFV clone 13 is an attenuated strain of RVFV, it can induce severe lesions in IFNAR^−/−^ and WT mice after intranasal infection. While IFN-I competence is of pivotal relevance for the prevention of RVFV viremia and subsequent hepatitis, the data demonstrate that RVFV can overcome an intact IFNAR signaling pathway in the CNS and induce fatal encephalitis. Furthermore, the experiments revealed an unexpected lack of encephalitis in surviving IFNAR^−/−^ mice that generally have a well-established high susceptibility to virus infection [38,39,53,64]. However, follow-up studies are needed to tackle the limitations of the present study and further elucidate the formation and pathogenesis of RVFE. Especially the investigation of intermediate sampling points up to 14 dpi, other RVFV strains and cell-type specific knockouts of the IFN-I system will critically contribute to the understanding of the immune response during RVFE on a cellular level.

## 4. Materials and Methods

### 4.1. Animal Experiment

Mice were bred under specific pathogen-free conditions in the central mouse facility of the Helmholtz Centre for Infection Research, Braunschweig, and at TWINCORE, Centre for Experimental and Clinical Infection Research, Hannover, Germany. Mice were kept at the TWINCORE, Centre for Experimental and Clinical Infection Research, a joint venture between the Helmholtz Centre for Infection Research and the Hannover Medical School, Hannover, Germany for the animal study and were fed a commercially available diet and water ad libitum. Animal handling was conducted under BSL-2 conditions.

All mice used in this study are on the C57BL/6 background and IFNAR^−/−^ mice were more than 20-fold backcrossed to the C57BL/6 background. Groups of ten male and female, 7–15 weeks old C57BL/6 WT mice (commercially obtained from Janvier) or IFNAR^−/−^ mice [38,64] were anesthetized by intraperitoneal injection of Ketamine/Xylazine, and intranasally infected with 10^3^ plaque-forming units (PFU) RVFV clone 13 (kindly provided by Dr. Marie Flamand, Institute Pasteur, Paris, France) or virus-free cell culture medium (mock infection). Moreover, five WT mice were infected intranasally with 10^4^ PFU RVFV clone 13. Clinical evaluation included the categories body weight, motility/behavior, and posture/clinical appearance and was carried out twice daily for 21 dpi. The 5–10% body weight loss, reduced spontaneous movement, and curled/ruffed fur were graded as mild signs. The 10–20% body weight loss, reduced provoked movement, and mildly curved spine were graded as moderate signs, whereas severe signs of disease were defined as >20% body weight loss, stupor, and severely curved spine and hunched posture or paralysis.

After 21 days or if severe signs in one category or moderate signs in all categories were present, animals were euthanized by intraperitoneal injection of an overdose of anesthesia and perfused with phosphate buffered saline. Brain, liver, and spleen samples were obtained for deep freezing. Additional organ samples (brain, spinal cord, spleen, thymus, liver, stomach, intestines, heart, lung, kidneys, cervical lymph nodes, sciatic nerves, skeletal muscles, and nose) were routinely fixed with 10% neutral-buffered formalin and embedded within paraffin wax. Prior to embedding, bone-containing tissues (nose and spinal cord samples with adjacent vertebra) were decalcified using 10% EDTA solution. Furthermore, blood samples were taken during infection (retro-bulbar sampling) and necropsy (cardiac blood). All animal experiments were performed in accordance with the respective authorities, local animal welfare officers and the German animal welfare law. Permission was granted by the Lower Saxony State Office for Consumer Protection and Food Safety (LAVES; permission number 33.19-42502-04-19/3323).

### 4.2. RNA Isolation and RT-qPCR

Frozen samples of liver and brain tissue were homogenized using the Qiagen tissue lyser II (Qiagen, Hilden, Germany) and viral RNA was isolated using the RNeasy Lipid Tissue Kit (Qiagen). RT-qPCR for RVFV was performed as described previously [65].

### 4.3. Histology, Immunohistochemistry and Immunofluorescence

Two to three µm thick paraffin sections were routinely stained with hematoxylin and eosin (HE) for histological evaluation. For detection of intralesional deposition of extracellular matrix (ECM) components, histochemical stainings including azan and picrosirius red were applied on spinal cord, brain, and liver sections as already described, with minor modifications including an extension of the incubation time with picrosirius red and omitting the nuclear counterstain in the azan staining [66,67]. Additionally, a modified alcian blue staining was used to identify formation of perivascular edema within brain and spinal cord [68]. Furthermore, immunohistochemistry was carried out using antibodies directed against RVFV, Iba-1, CD3, CD45R, entactin/nidogen-1, GFAP, laminin, and cleaved caspase-3 (Table 2), biotinylated rabbit anti-sheep and goat anti-rabbit secondary antibodies (1:200; Vector Laboratories, Burlingame, CA, USA) and the avidin-biotin-peroxidase-complex (ABC) method (PK-6100; Vector Laboratories) with 3,3′-diaminobenzidine tetrahydrochloride (DAB; Sigma-Aldrich, St. Louis, MO, USA) as chromogen [69,70].

Double staining immunofluorescence for the simultaneous detection of neurofilament and RVFV nucleoprotein was performed using respective primary antibodies (Table 2), followed by incubation with Cy2 (donkey anti-mouse)- and Cy3 (donkey anti-sheep)-conjugated secondary antibodies (Jackson Immunoresearch, Philadelphia, USA; 1:200) according to previously published protocols for formalin-fixed, paraffin-embedded material [71].

Longitudinal brain sections were evaluated in 11 areas including the olfactory bulb, cerebral cortex/corpus callosum/anterior olfactory nucleus, caudoputamen, ventral striatum/basal forebrain, hippocampus, thalamus, hypothalamus, quadrigeminal lamina, ventral midbrain, cerebellum, and pons/medulla oblongata (Figure S4). Lesions were scored using a semiquantitative scoring system for meningitis (0: normal; 1: single inflammatory cells; 2: 2–3 layers of inflammatory cells; 3: >3 layers of inflammatory cells). To quantify cellular necrosis, slides were scanned (Olympus^®^ Slideview VS200) and areas were measured using Olympus^®^ OlyVIA 3.2 (0: normal; 1: 1–25%; 2: 26–50%; 3: >50% of cells necrotic in one area). Immunohistochemistry was evaluated descriptively via light microscopy by two different pathologists.

### 4.4. Statistical Analysis

Statistical analysis (Mantel–Cox test, Fisher’s *t*-test and Kruskal–Wallis test with Dunn’s multiple comparisons tests, two-way-ANOVA and Sidaks multiple comparisons test) was performed using GraphPad Prism 9.0 (GraphPad Software, San Diego, CA, USA). Differences between groups were considered significant at *p*-values of <0.05.

## Figures and Tables

**Table 1:** Histological findings and immunohistochemical detection of RVFV nucleoprotein.

	WT				IFNAR^−/−^	
Infection Dose	10^4^ PFU (n = 5, 7–21 dpi) *		10^3^ PFU (n = 10, 21 dpi)		10^3^ PFU (n = 10, 2–21 dpi) **	
Staining	HE	RVF-Ag	HE	RVF-Ag	HE	RVF-Ag
Brain	multifocal neuronal necrosis of varying severity (n = 4) multifocal mild (n = 2) to moderate (n = 2) leptomeningitis	many neurons (n = 4)	focal mild meningitis (n = 2)	n.d.	focal mild neuronal necrosis (n = 1) multifocal mild meningitis (n = 2)	few neurons (n = 1)
Spinal cord	multifocal mild neuronal necrosis (n = 2)	many neurons (n = 2)	n.s.m.l.	n.d.	n.s.m.l.	n.d.
Liver	n.s.m.l.	n.d.	n.s.m.l.	n.d.	diffuse hepatocellular necrosis (n = 5)	many hepatocytes (n = 5)
Nose	n.s.m.l.	n.d.	n.s.m.l.	n.d.	multifocal mild necro-suppurative rhinitis	single cells in olfactory and respiratory mucosa (n = 1)
Spleen	n.s.m.l.	n.d.	n.s.m.l.	n.d.	multifocal lymphocyte necrosis (n = 5)	many lymphocytes (n = 5)
Blood vessels	n.s.m.l.	n.d.	n.s.m.l.	n.d.	n.s.m.l.	many monocytes (n = 5)
Bone marrow	n.s.m.l.	n.d.	n.s.m.l.	n.d.	n.s.m.l.	many hematopoietic precursor cells (n = 5)

* lesions were found between 7 and 11 dpi. ** lesions were found between 2 and 5 dpi. dpi: days post infection; Ag: antigen; HE: hematoxylin and eosin; RVFV: Rift Valley fever virus; PFU: plaque- forming units; n.s.m.l.: no significant microscopic lesions; n.d.: not detected. No significant lesions or RVFV antigen were detected in mock-infected wild type (WT) and type I interferon receptor knockout (IFNAR^−/−^) mice.

**Table 2:** Primary antibodies used for immunohistochemistry.

Antigen	Host Species	Pretreatment	Dilution	Supplier
CD3 (T lymphocyte)	rabbit	microwave, citrate buffer, 20 min	1:2000	DakoCytomation, Hamburg, Germany
CD45R (B lymphocyte)	rat	microwave, citrate buffer, 20 min	1:1000	Becton Dickinson, Franklin Lakes, USA
Cleaved caspase 3 (apoptotic cells)	rabbit	microwave, citrate buffer, 20 min	1:200	Cell Signaling Technology, Cambridge, UK
Entactin/Nidogen-1	rabbit	microwave, citrate buffer, 20 min	1:3000	Abcam, Cambridge, UK
GFAP (astrocyte)	rabbit	None	1:1000	DakoCytomation, Hamburg, Germany
Iba-1 (macrophage/microglia)	rabbit	microwave, citrate buffer, 20 min	1:1000	Wako Chemicals, Richmond, USA
Laminin	rabbit	None	1:50	Sigma-Aldrich Chemie GmbH, Taufkirchen, Germany
Non-phosphorylated neurofilament	mouse	microwave, citrate buffer, 20 min	1:2000	BioLegend, San Diego, USA
RVFV nucleoprotein	sheep	None	1:50,000	Friedrich-Loeffler-Institute, Greifswald, Germany [70]

CD: cluster of differentiation; GFAP: glial fibrillary acidic protein; Iba-1: ionized calcium-binding adapter molecule 1; RVFV: Rift Valley fever virus.

## Data Availability

The data presented in this study are not publicly available but are available upon reasonable request.

## Supplementary Materials

The following supporting information can be downloaded at: https://www.mdpi.com/article/10.3390/ijms232012492/s1.
