# Supplementary material for: Intact Type I Interferon Receptor Signaling Prevents Hepatocellular Necrosis but Not Encephalitis in a Dose-Dependent Manner in Rift Valley Fever Virus Infected Mice
Source: Int J Mol Sci. 2022 Oct 18;23(20):12492. doi: 10.3390/ijms232012492 (PMC9603964; doi:10.3390/ijms232012492)
Supplement: Supplementary file 1 [file ijms-23-12492-s001.zip › ijms-1893589-supplementary.pdf]

Supplementary Files

Intact Type I Interferon Receptor Signaling Prevents Hepatocellular Necrosis but Not Encephalitis in a Dose-Dependent Manner in Rift Valley Fever Virus Infected Mice

Lukas Mathias Michaely, Lukas Schuwerk, Lisa Allnoch, Kathleen Schön, Inken Waltl, Pia-Katharina Larsen, Andreas Pavlou, Chittappen Kandiyil Prajeeth, Guus F. Rimmelzwaan, Stefanie C. Becker, Ulrich Kalinke, Wolfgang Baumgärtner and Ingo Gerhauser

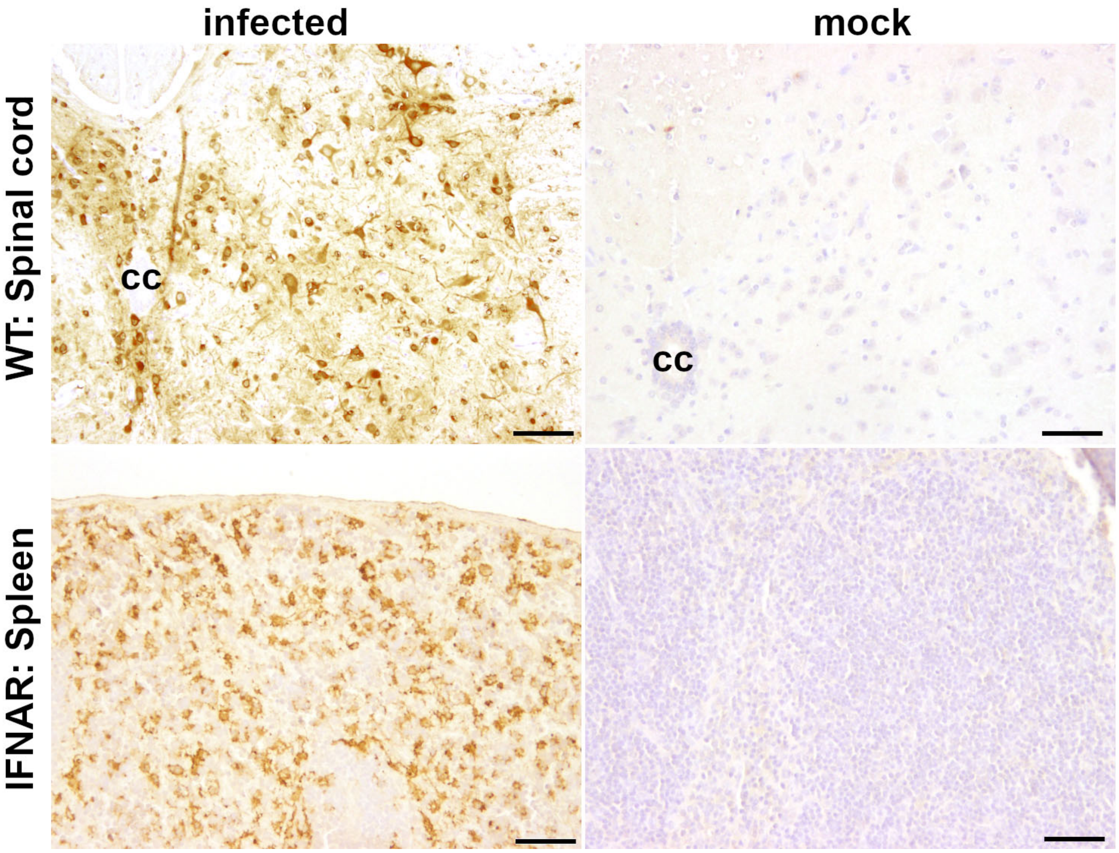

Supplementary Figure S1. Detection of Rift Valley fever virus (RVFV) antigen in the spinal cord and spleen. Virus antigen is found in spinal cord neurons of a wild type (WT) mouse infected with 10^4 plaque-forming units (PFU) RVFV at 9 days post infection (dpi) and in the spleen (5 dpi) of a type I interferon receptor knockout (IFNAR$^{-/-}$) mouse infected with 10^3 PFU RVFV. Lack of viral antigen in mock-infected animals (WT and IFNAR$^{-/-}$) at 21 dpi. Immunohistochemistry using a polyclonal antibody directed against RVFV, the avidin-biotin-peroxidase-complex (ABC) method and 3,3′-diaminobenzidine tetrahydrochloride (DAB) as chromogen. cc = central canal. Bars = 100 μm.

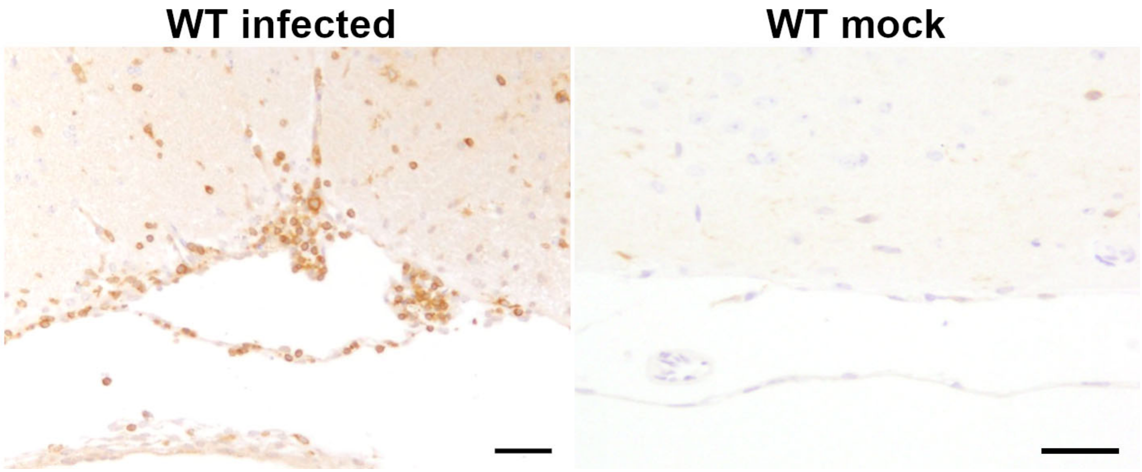

Supplementary Figure S2. Moderate numbers of CD3$^+$ T cells in the meninges of the hypothalamus of a wild type (WT) mouse infected with 10^4 PFU Rift Valley fever virus at 11 days post infection (dpi). No CD3$^+$ T cells were found in a mock-infected WT mouse at 21 dpi. Immunohistochemistry using a polyclonal antibody directed against CD3, the avidin-biotin-peroxidase-complex (ABC) method and 3,3′-diaminobenzidine tetrahydrochloride (DAB) as chromogen. Bars = 50 μm.

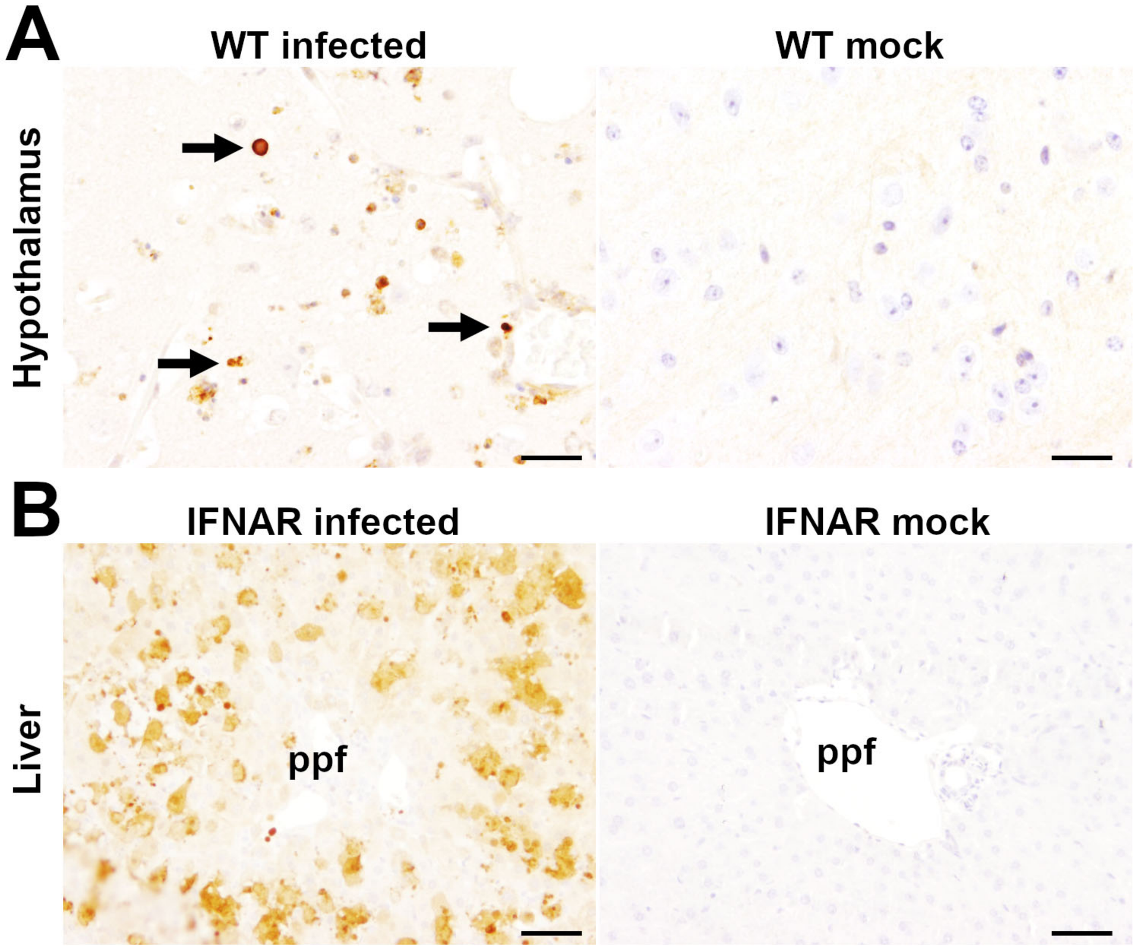

Supplementary Figure S3. Detection of apoptotic cells characterized by cleaved caspase 3 immunoreactivity in the hypothalamus and liver of wild type (WT) and type I interferon receptor knockout ($IFNAR^{-/-}$) mice infected with Rift Valley fever virus (RVFV). Shown are cleaved caspase 3 positive cells (arrows) in the hypothalamus of a WT mouse infected with 10^4 plaque-forming units (PFU) (**A**, 11 dpi) and the liver of a $IFNAR^{-/-}$ mouse infected with 10^3 PFU (**B**, 5 dpi). Absence of cleaved caspase 3 positive cells in the hypothalamus of a mock-infected WT and the liver of a mock-infected $IFNAR^{-/-}$ mice at 21 dpi. Immunohistochemistry using a polyclonal antibody directed against cleaved caspase-3, the avidin-biotin-peroxidase-complex (ABC) method and 3,3′-diaminobenzidine tetrahydrochloride (DAB) as chromogen. ppf = periportal field. Bars**:** Hypothalamus = 50 μm; liver = 100 μm.

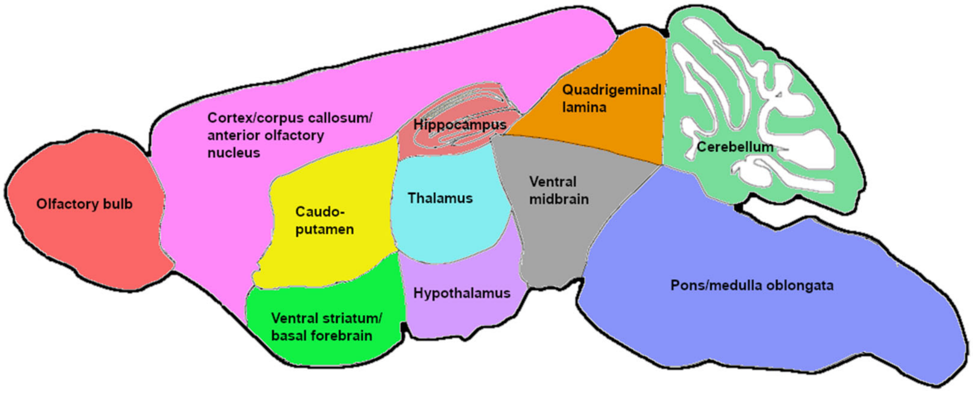

Supplementary Figure S4. Overview of histologically investigated brain area distribution. Longitudinal brain sections were scored semiquantitatively in the following areas: 1. Olfactory bulb; 2. Cerebral cortex/corpus callosum/anterior olfactory nucleus; 3. Caudoputamen; 4. Ventral striatum/basal forebrain; 5. Hippocampus; 6. Thalamus; 7. Hypothalamus; 8. Quadrigeminal lamina; 9. Ventral midbrain; 10. Cerebellum; 11. Pons/medulla oblongata.
